# *Salmonella* Outer Protein B Suppresses Colitis Development via Protecting Cell From Necroptosis

**DOI:** 10.3389/fcimb.2019.00087

**Published:** 2019-04-09

**Authors:** Gui-Qiu Hu, Yong-Jun Yang, Xiao-Xia Qin, Shuai Qi, Jie Zhang, Shui-Xing Yu, Chong-Tao Du, Wei Chen

**Affiliations:** Key Laboratory of Zoonosis Research, Ministry of Education, College of Veterinary Medicine, Jilin University, Changchun, China

**Keywords:** *Salmonella*, SopB, colitis, MLKL, necroptosis

## Abstract

*Salmonella* effectors translocated into epithelial cells contribute to the pathogenesis of infection. They mediate epithelial cell invasion and subsequent intracellular replication. However, their functions *in vivo* have not been well-identified. In this study, we uncovered a role for *Salmonella* outer protein B (SopB) in modulating necroptosis to facilitate bacteria escape epithelial cell and spread to systemic sites through a *Salmonella*-induced colitis model. Mice infected with SopB deleted strain Δ*sopB* displayed increased severity to colitis, reduced mucin expression and increased bacterial translocation. *In vitro* study, we found there was an increased goblet cell necroptosis following Δ*sopB* infection. Consistently, mice infected with Δ*sopB* had a strong upregulation of mixed lineage kinase domain-like (MLKL) phosphorylation. Deletion of MLKL rescued severity of tissue inflammatory, improved mucin2 expression and abolished the increased bacterial translocation in mice infected with Δ*sopB*. Intriguingly, the expression of *sopB* in LS174T cells was downregulated. The temporally regulated SopB expression potentially switched the role from epithelial cell invasion to bacterial transmission. Collectively, these results indicated a role for SopB in modulating the onset of necroptosis to increased bacteria pathogenesis and translocated to systemic sites.

## Introduction

*Salmonella* enterica is a leading cause of enteric disease in human and animals that is capable of causing a wide range of illnesses ranging from a localized gastroenteritis and lymphadenitis resulting in diarrhea to life-threatening systemic infections (Kirk et al., 2015). *Salmonella* has evolved many mechanisms to evade host immune response to survive in host cell via virulence effectors (Tobar et al., 2006; McGhie et al., 2009; Raymond et al., 2013). *Salmonella* has two major virulence determinants called *Salmonella* pathogenicity island (SPI)-1 and SPI-2 (Hensel, 2004). SPI-1 is essential for invasion of non-phagocytic cells and responsible for inflammation in *Salmonella* induced colitis, whereas SPI-2 is required for intracellular survival and proliferation in phagocytes, and is important in systemic infection (Barthel et al., 2003; Abrahams and Hensel, 2006). Both SPI-1 and SPI-2 encode separated type III secretion systems (T3SSs) that direct the translocation of virulence proteins into host environment (Galan, 2001; Schmidt and Hensel, 2004). These effector proteins modulate host cell immunity and are important for bacterial pathogenesis.

*Salmonella* outer protein B (SopB) is SPI-1 encoded protein. Its synthesis is tightly regulated. The long half-life in cells allows SopB to exert multiple roles during *Salmonella* infection (Giacomodonato et al., 2011). SopB is responsible for membrane ruffle formation and subsequent invasion (Piscatelli et al., 2016). In addition to its role in invasion, numerous biological roles have been explored attributing to its inositol phosphatase activity. For instance, SopB participates in modulation of chloride secretion (Bertelsen et al., 2004) and Akt phosphorylation (Cooper et al., 2011). Studies have shown that SopB protects epithelial cell from apoptosis (Knodler et al., 2005; Ruan et al., 2016). Additionally, SopB mutation mediated increased macrophage death results in enhanced inflammasome activation in inositol phosphatase activity dependent manner (Hu et al., 2017). The role of SopB in protecting cells from death represents a bacterial strategy to reduce host response.

Cells death mediated by *Salmonella* infection is considered to be an important pathological process and a major cause of tissue damage during infection. Initial studies showed that *Salmonella* induce cell apoptosis (Knodler et al., 2005), however, recent studies demonstrate that cell death can be mediated through many different ways such as necroptosis and pyroptosis (Broz et al., 2010; Hefele et al., 2018). Apoptosis is a non-lytic programmed cell death which is usually immunological silent, while necroptosis and pyroptosis are lytic cell death and usually result in inflammatory response. Many effector proteins have involved in regulating epithelial cell death which is considered a mechanism used by *Salmonella* to escape epithelial cell and spread to systemic sites. Previous studies showed SopB protects cell from death in mechanisms of apoptosis and/or pyroptosis (Knodler et al., 2005; Hu et al., 2017), however, the mechanisms of SopB *in vivo* has not been well-studied.

In this study, we used a *Salmonella* induced colitis model to evaluate the role of SopB in bacterial pathogenesis and employed SopB mutant strain Δ*sopB* as well as mixed lineage kinase domain-like (MLKL) deficient mice to elucidate the contribution of SopB in manipulating host immune response during bacterial invasion and dissemination *in vivo*.

## Materials and Methods

### Animals

C57BL/6J (WT) and MLKL deficient (MLKL^−/−^) mice, on a C57BL/6J background, were bred in house. Mice were maintained under conditions of a 12 h light/dark cycle at 23°C with food and water *ad libitum* in the animal care facility. All animal experiments were performed in accordance with the relevant guidelines and regulations that were approved by the Committee on Animal Care and Use of Jilin University, China.

### *In vivo* Infection

Six- to eight- weeks old and sex-matched mice were used in this study. The *Salmonella*-induced colitis model was established as previously described (Stecher et al., 2005). Briefly, following the administration of 20 mg streptomycin per mouse for 24 h, mice were orally challenged with 5 × 10^7^ colony-forming unit (CFU) of *Salmonella* strain SL1344 or SopB deleted strain Δ*sopB*. Bacterial burdens and histopathology of cecum were determined at indicated time point post infection (p.i.).

### Cell Culture

LS174T human goblet-like cells were cultured in DMEM/F12 (Gibco, Waltham, MA, USA) containing 10% heat inactivated fetal bovine serum. Cells were seeded in 24-well dish at 0. 3 × 10^6^ cells per well and cultured overnight to yield monolayers of 80–90% confluence. Cells were pretreated with or without the MLKL inhibitor NSA (Toronto Research Chemical Inc., Toronto, Ontario, Canada) at a final concentration of 2 nM or the receptor-interacting protein kinase (RIP1) inhibitor Nec-1(Tocris, Minneapolis, MN, USA) at a final concentration of 50 μM for 1 h. *Salmonella* strains were then inoculated to LS174T cells at a multiplicity of infection = 100 for 1 h, then the extracellular bacteria were removed by washing with DMEM/F12. Cells were then maintained in DMEM/F12 containing 200 U/mL streptomycin and 100 μg/mL gentamicin for another 3 h, cell viability was evaluated. For mucin-2 examination, cells were seeded in 6-well dish at 1.5 × 10^6^ cells per well and cultured overnight, then infected with *Salmonella* strains for 14 and 24 h respectively and lysed in lysis buffer.

### Dot Blot Assay

Add 20 ug total protein to NC membranes (0.45 μm, Merck Millipore, Darmstadt, Germany) and let the membrane dry at room temperature (RT). Blocking non-specific sites by soaking in 5% BSA in Tris buffered saline containing 0.1% (v/v) Tween 20 (Sigma-Aldrich, St. Louis, MO, USA) (TBST) for 2 h at RT and then incubating the membranes with primary antibody anti-Mucin2 (1:500, Santa Cruz, CA, USA) for 1 h at RT. Wash the membrane with TBST for 3 times, each time for 10 min, and then incubate with secondary antibody for 1 h at RT.

### Histology and Immunohistochemisty (IHC)

On day 2 p.i., the cecum was harvested and fixed in 4% paraformaldehyde and then embedded in paraffin; 5 μm sections were used for hematoxylin-eosin (H&E) staining and IHC staining. The assessment of tissue pathology was scored as previously described (Stecher et al., 2005). For IHC staining, sections were dewaxed and rehydrated, and antigen was unmasked in a citrate-containing buffer, and then immersed the sections in 3% hydrogen peroxide for 10 min, blocked the non-specific antigen with 5% goat serum for 1 h at RT, then incubated with the primary antibody anti-Mucin2 (1:100, Santa Cruz, CA, USA) and anti-p-MLKL (1:100, AbCam, Cambridge, MA, USA) at 4°C overnight respectively, then cover sections with detection antibody.

### PAS Staining

After IHC staining, sections were oxidized in periodic acid solution (Solarbio, Beijing, China) for 5 min and then rinsed in distilled water. Subsequently, sections were immersed in Schiff reagent (Solarbio, Beijing, China) for 15 min, and then rinsed in distilled water.

### Total RNA Extraction and Quantitative Real-Time PCR

Total RNA was extracted from LS174T cells and cecal tissue samples at indicated time point using TRI-Reagent (Sigma-Aldrich, St. Louis, MO, USA) according to the manufacturer's guidelines. Bacterial RNA was isolated following manufacturer's instructions (Promega, Madison, WI, USA). cDNA was synthesized using Rtase M-MLV (TaKaRa, Kyoto, Japan) with oligo (dT). Quantitative real-time PCR was performed on an ABI 7500 Fast Real-Time PCR System (Applied Biosystems) using SYBR Green PCR Master Mix (Roche, South San Francisco, CA, USA). GAPDH was used as the reference gene. The relative changes in gene expression were analyzed by the 2^−ΔΔct^ method. The relative primers are listed in Table 1.

**Table 1:** Primers used for qRT-PCR.

**Gene**	**Sequence**
Mouse Mucin2	F:5′-GCTGACGAGTGGTTGGTGAATG-3′
	R:5′-GATGAGGTGGCAGACAGGAGAC-3′
Mouse GAPDH	F:5′-AGGTCGGTGTGAACGGATTTG-3′
	R:5′-GGGTCGTTGATGGCAACA-3′
Human Mucin2	F:5′-GAGGGTGGAAGTGGCAT-3′
	R:5′-TGTCGGCAGGGTTGA-3′
Human GAPDH	F:5′-ACATCATCCCTGCCTCTACTG-3′
	R:5′-ACCACCTGGTGCTCAGTGTA-3′
SopB	F:5′-GGAATTGTAAAAGCGGCAAA-3′
	R:5′-TTTTCTGTCCACCGCTATCC-3′
SopE_2_	F:5′-GGAGAGGTTATGCCGCCTTT-3′
	R:5′-CGGAGTGATCCTCAAGGCAA-3′
Eubacteria (Universal)	F:5′-ACTCCTACGGGAGGCAGCAGT-3′
	R:5′-ATTACCGCGGCTGCTGGC-3′

### Cytotoxicity Assays

Cell viability was evaluated by the measurement of lactate dehydrogenase (LDH) leakage from damage or destroyed cells with a CytoTox 96 Non-Radioactive Cytotoxicity Assay Kit under the manufacturer's instruction (Promega, Madison, WI, USA).

### Bacterial Counts

Cecum of mice were flushed free of feces with PBS. Tissues including liver, spleen, MLN and cecum of mice were weighted and then homogenized in cold PBS at a ratio of 1:6 (g/mL). The homogenates were serial diluted and 50 ul was plated on LB plates containing streptomycin. After 16 h, colonies were counted.

### Cytokines Measurement

Cecum of mice were harvested and flushed free of feces with PBS, and then weighted and homogenized in cold PBS at a ratio of 1:6 (g/mL). Homogenates were centrifuged at 13000 rpm/min for 30 min and supernatants were collected and used for cytokines measurement. Cytokines were detected with ELISA assay following R&D systems instruction.

### Statistical Analysis

All experiments were independently performed three times in triplicate. All values were expressed as means ± standard deviations. Differences between mean values were assessed by ANOVA test. The analysis of survival was determined via log-rank test. Data analysis was performed with GraphPad Prism software version 6.0 (GraphPad, La Jolla, CA, USA). *P* value was < 0.05 was considered to be significant different. *P < 0.05, **P < 0.01, ***P < 0.001, #*P* < 0.05, ##*P* < 0.01.

## Results

### Mice Display Increased Susceptibility to Δ*sopB* Induced Colitis

**Figure 1:**
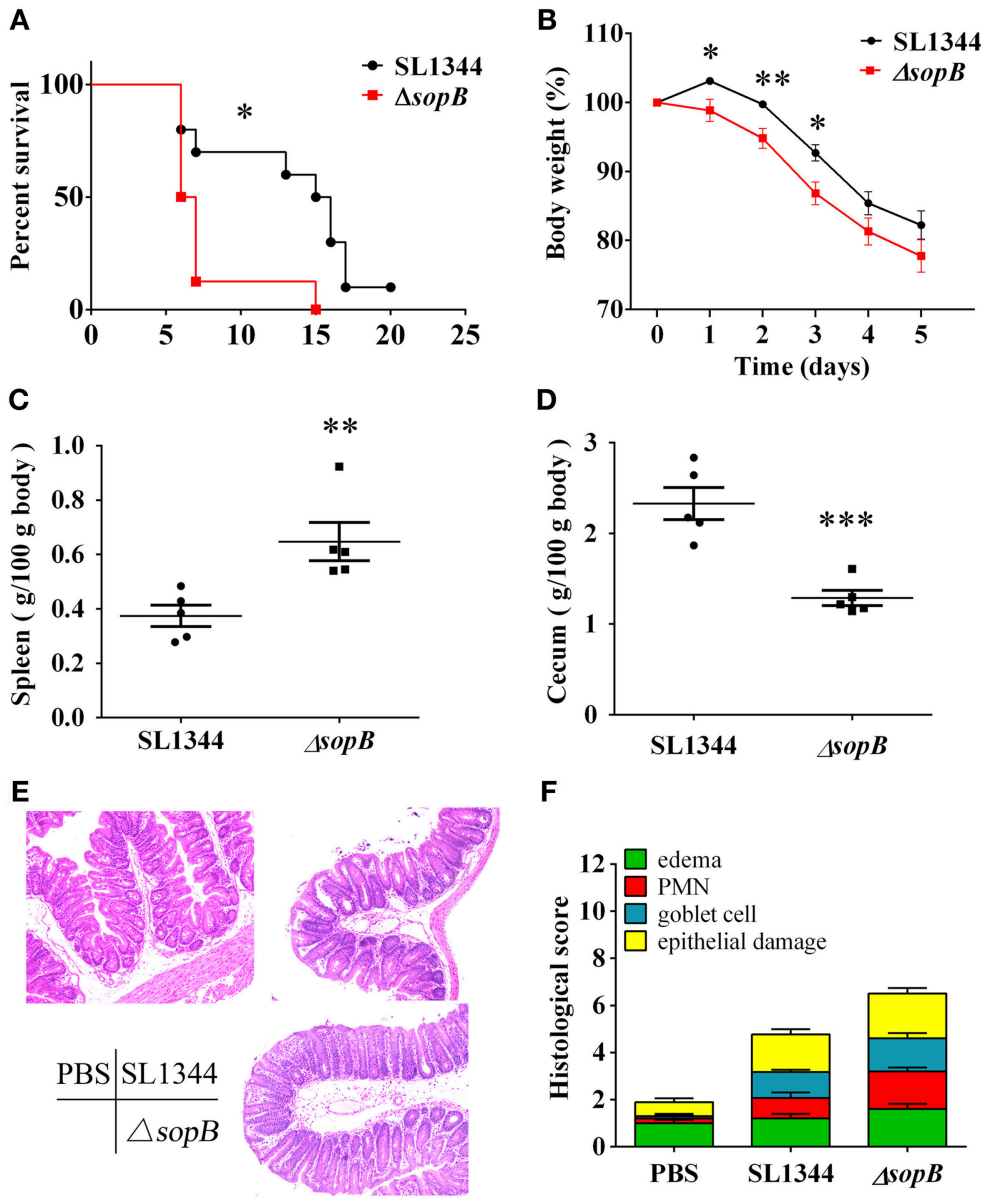
Δ*sopB* infection induced increased severity to colitis. Mice were treated with streptomycin prior to oral administration of SL1344 or Δ*sopB*, **(A)** Survival (*n* = 10); **(B)** Body weight change (*n* = 5); **(C)** Spleen weight on day 2 p.i. (*n* = 5); **(D)** Cecum weight on day 2 p.i. (*n* = 5); **(E)** Representative H&E staining of cecal tissues harvested from mice orally administrated with PBS, SL1344 or Δ*sopB* for 2 days (*n* = 5), magnification, ×100; **(F)** Pathological score for the cecal samples. *P*-value for survival was determined via log-rank test. *P < 0.05, **P < 0.01, ***P < 0.001.

Although SopB mediates sustained activation of the pro-survival kinase Akt in infected epithelial cells (Knodler et al., 2005), and moreover, Akt2 deficiency mice display increased susceptibility to *Salmonella* infection (Kum et al., 2011), the role of SopB *in vivo* is not clear to now. We hypothesized that SopB deleted SL1344 strain Δ*sopB* would affect the host's defense against *Salmonella* infection, leading to increased severity to disease. Therefore, we initially examined the susceptibility to colitis following the oral administration of 5 × 10^7^ colony-forming units (CFU) of *Salmonella* strain SL1344 or SopB mutant strain Δ*sopB*. Compared with SL1344 infection, Δ*sopB* infected mice displayed significant higher mortality (Figure 1A) and increased body weight loss (Figure 1B). Mice infected with Δ*sopB* displayed a significant increased splenomegaly (Figure 1C) and decreased cecum weight (Figure 1D) when compared with SL1344 infection. Consistently, H&E staining showed Δ*sopB* infected mice had increased intestinal damage and histopathology score characterized by greater submucosa edema, elevated polymorphonuclear (PMN) leukocyte infiltration, severe mucosa ulceration and increased goblet cells depletion (Figures 1E,F). Collectively, these data indicated that SopB plays a redundant role in the induction of intestinal inflammation during *Salmonella* infection, which deletion increased severity to colitis.

### Δ*sopB* Infection Leads to Reduced Mucin Expression

**Figure 2:**
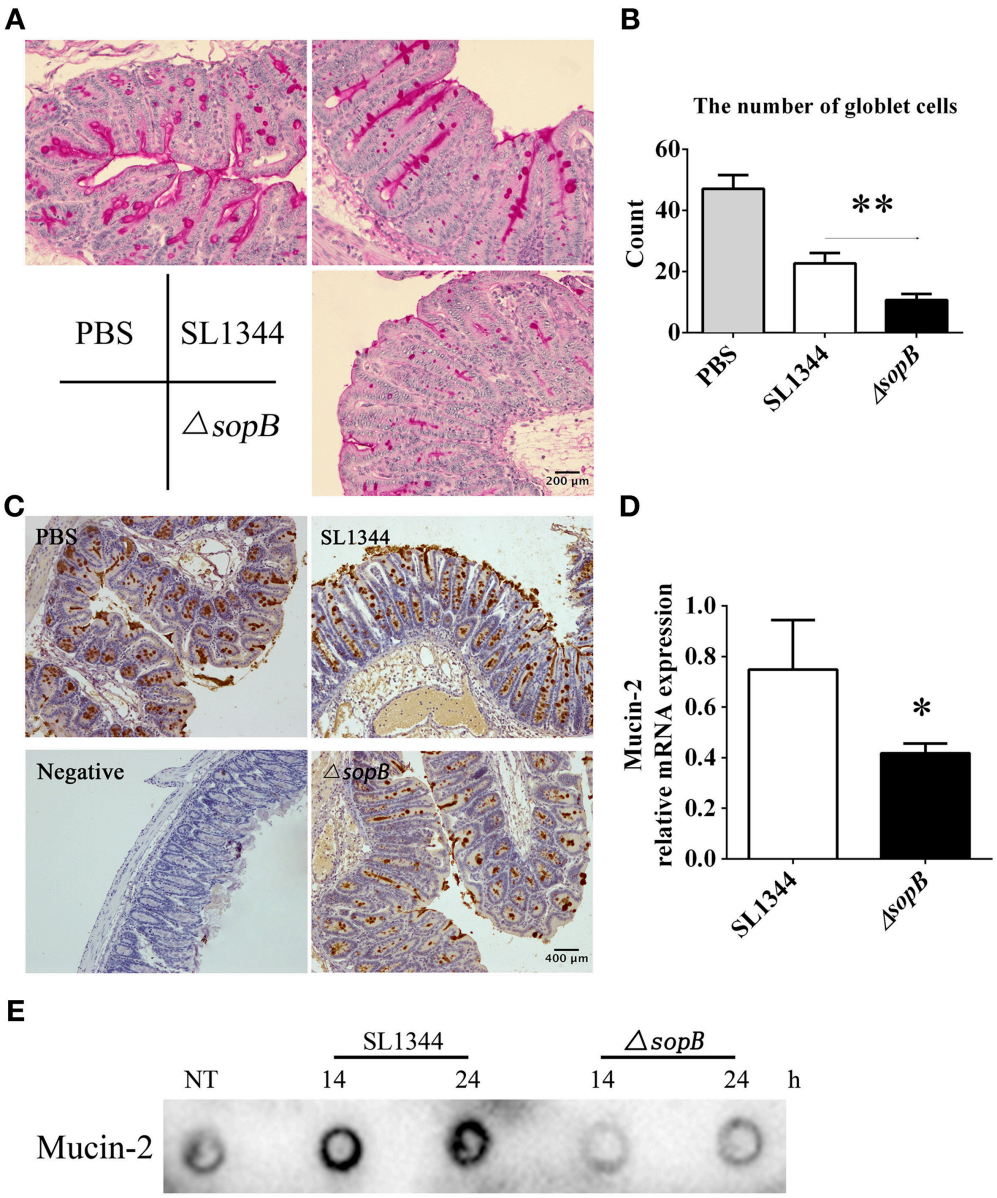
Mice infected with Δ*sopB* had a disturb goblet cell function. Mice were orally administrated with PBS, SL1344 or Δ*sopB* for 2 days (*n* = 5), **(A)** Total mucins expression in goblet cells were examined via PAS staining; **(B)** The average number of goblet cells per high-power field (magnification, × 400) was calculated from 10 different regions of the cecal epithelium; **(C)** Mucin2 expression in cecum was determined via IHC staining; **(D)** The mRNA level of mucin2 in cecum was examined by real-time PCR, the data was normalized to GAPDH expression and showed as the fold increase in mRNA. **(E)** The expression of mucin2 in LS174T cells treated with SL1344 or Δ*sopB* for 14 and 24 h respectively. *P < 0.05, **P < 0.01.

The major difference of histopathology in cecum between SL1344 and Δ*sopB* infection was especially in goblet cell depletion (Figure 1F). Goblet cells exert numerous biological roles to protect the gut, such as mucin secretion and antigens delivering (Pelaseyed et al., 2014; Birchenough et al., 2016). Mucus serves as the first physical barrier encountered by *Salmonella*. Therefore, mucins expression was further investigated. When compared with SL1344 infection, there was a significant reduction in mucin expression (Figure 2A) and numbers of mucin containing goblet cells (Figure 2B) in cecum following Δ*sopB* infection. Mucin2 is a major mucin secreted by goblet cells and has an important function in protecting against enteric pathogens (Bergstrom et al., 2010). The expression of mucin2 in cecum was subsequently examined. In concert with mucins expression, we found there was a significant reduction of mucin2 mRNA and protein in cecum following Δ*sopB* infection for 2 days (Figures 2C,D). Consist with the *in vivo* study, we also found there was a reduction in mucin2 expression in LS174T cells infected with Δ*sopB* for 14 and 24 h respectively, when compared with SL1344 infection (Figure 2E). These results indicated that Δ*sopB* infection results in significant reduction in goblet cells number and mucin expression.

### Δ*sopB* Infection Promotes Goblet Cells Necroptosis

**Figure 3:**
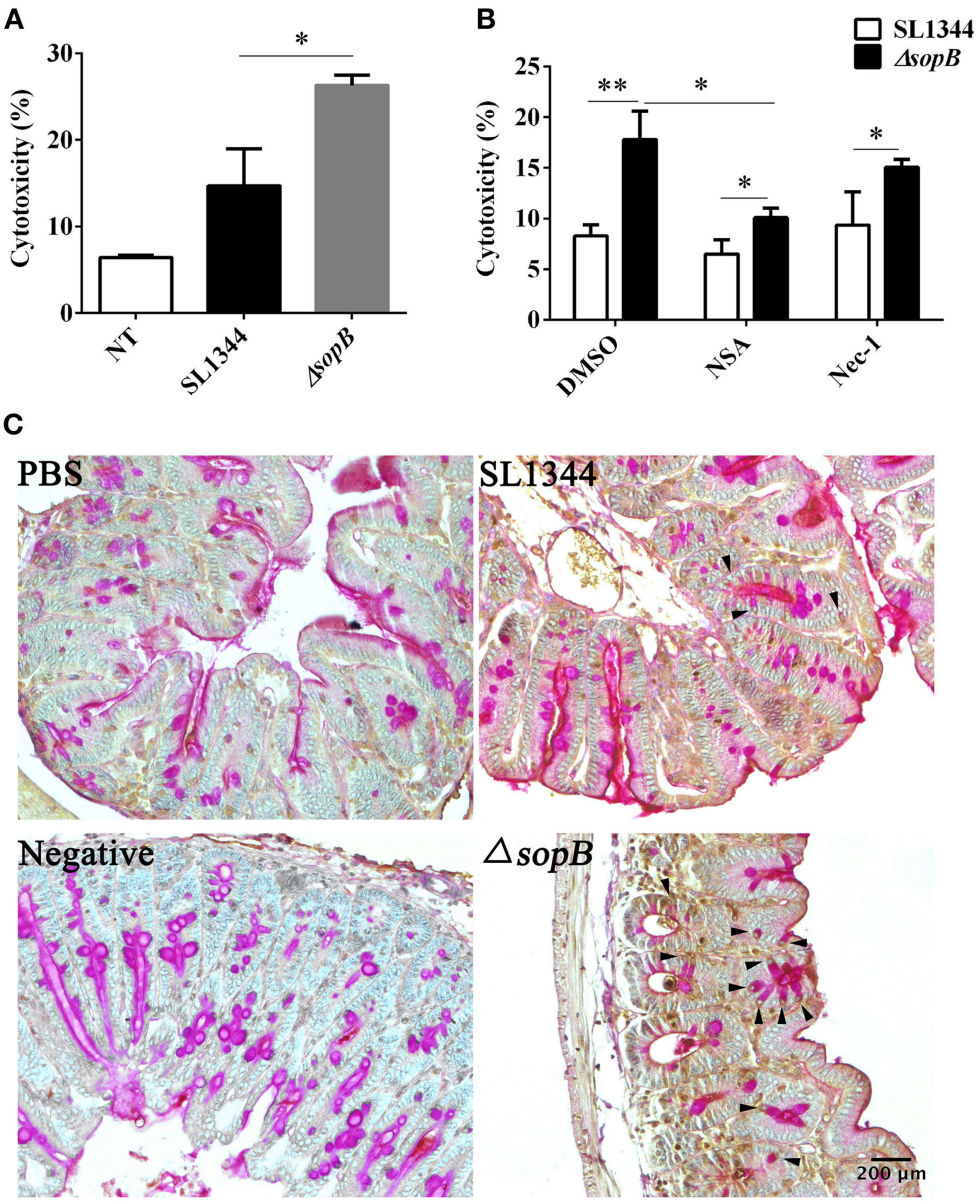
SopB protected goblet cells from necroptosis. **(A)** Cell death was determined via measuring the release of lactate dehydrogenase (LDH) from LS174T cells infected with SL1344 or Δ*sopB* for 4 h at a MOI = 100; **(B)** Determination of LDH released from LS174T cells pretreated with necroptosis inhibitor NSA or Nec-1 and then followed by SL1344 or Δ*sopB* infection; **(C)** The level of MLKL phosphorylation in cecum tissues was evaluated by IHC staining, and then tissues were counter stained by PAS staining to indicate MLKL phosphorylation in goblet cells, as the arrowhead showed. *P < 0.05, **P < 0.01.

The reduced mucin expression attributes to the disturbed goblet cell function. Thus, we subsequently examined whether there was an increased goblet cells death following Δ*sopB* infection. We found that Δ*sopB* infection induced increased LS174T cell death when compared with SL1344 infection (Figure 3A), however, whether the goblet cells went on death in a manner of necroptosis has not been investigated. To elucidate this question, LS174T cells were pretreated with a necroptosis inhibitor necrostatin-1 (nec-1) or NSA before bacterial infection. Surprisingly, treatment with NSA, an inhibitor of MLKL, significant reduced LS174T cell death following Δ*sopB* infection, however, had no obvious effect on SL1344 induced cell death (Figure 3B). These results indicated that Δ*sopB* infection can induce goblet cells death in a manner of necroptosis. The molecular nec-1 is a RIPK1 inhibitor which is upstream of MLKL. We found nec-1 treatment did not significantly reduce LS174T cell death following Δ*sopB* infection (Figure 3B). According to these results, we concluded that Δ*sopB* infection increased LS174T cell necroptosis via upregulating MLKL phosphorylation. To further confirm the observed phenomenon *in vitro*, we examined the level of MLKL phosphorylation in cecum following bacterial infection for 2 days. Consistent with the *in vitro* results, we found there was a significant increased expression of MLKL phosphorylation in goblet cells as well as in the other epithelial cells (Figure 3C). Collectively, these results indicated that Δ*sopB* infection promotes cell necroptosis via upregulating MLKL phosphorylation.

### MLKL Deletion Abolishes the Severe Inflammation in Cecum Following Δ*sopB* Infection

**Figure 4:**
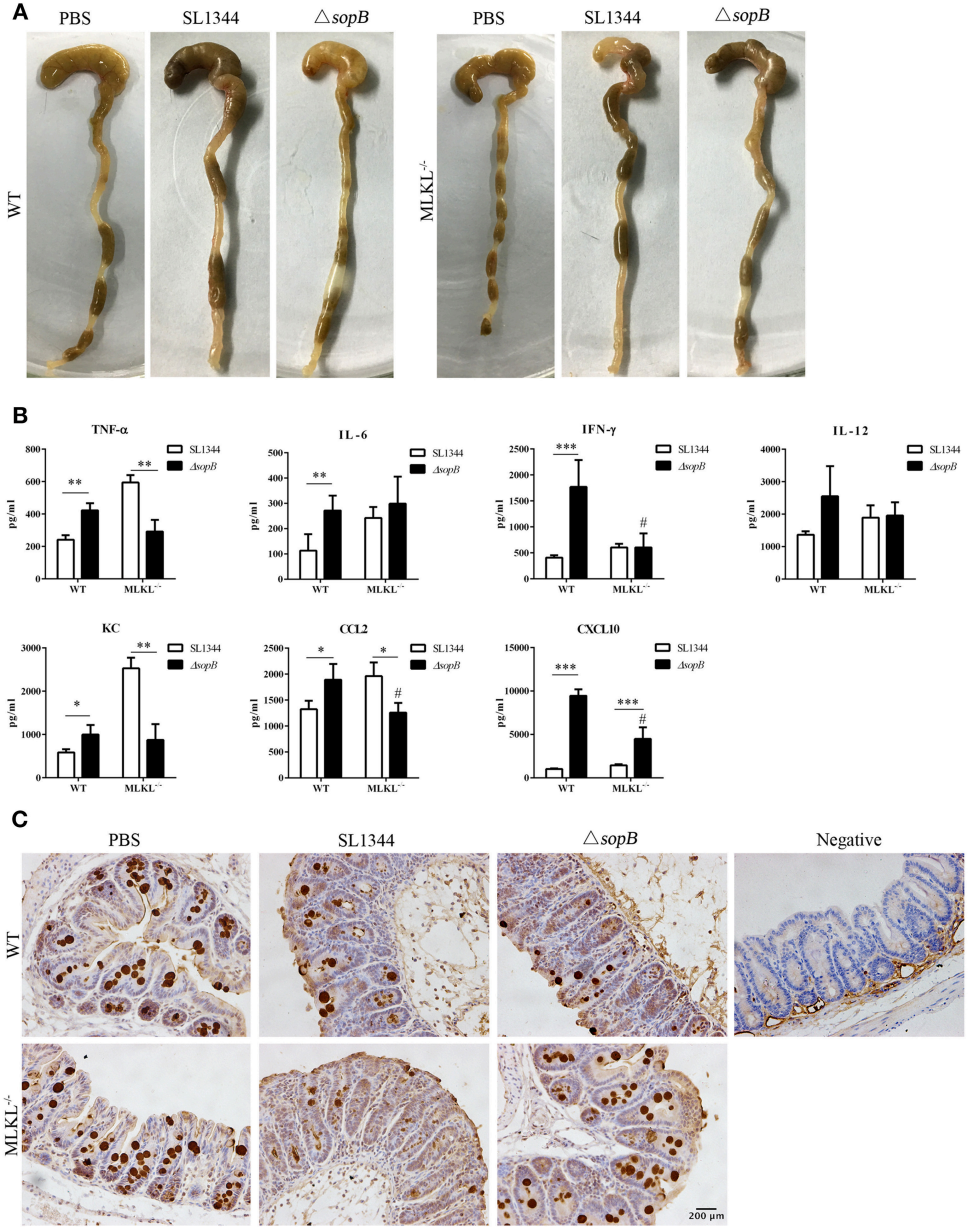
MLKL deletion abolished the increased inflammatory in mice infected with Δ*sopB*. Mice were orally administrated with PBS, SL1344 or Δ*sopB* for 2 days (*n* = 5), **(A)** Gross picture of cecum; **(B)** Inflammatory cytokine production in cecum of WT and MLKL^−/−^ mice; **(C)** Mucin2 expression in cecum. *P < 0.05, **P < 0.01, ***P < 0.01, ^#^*P* < 0.05; ^#^stands for comparing with WT mice infected with Δ*sopB*.

To further identify the contribution of necroptosis in Δ*sopB* mediated increased severity to colitis, we employed MLKL deficient mice. Intriguingly, MLKL deletion rescued severity to Δ*sopB* induced colitis. Following Δ*sopB* infection for 2 days, there was a significant decrease in the weight of cecum, however, MLKL deletion improved the loss of cecum weight (Figure 4A). Consistently, MLKL deletion also ameliorated the inflammatory cytokines production in cecum following Δ*sopB* infection (Figure 4B). There was a significant increased inflammatory cytokine production including TNF-α, IL-6, IFN-γ, KC, CCL2 and CXCL10, however, no significant difference was found in IL-12 production in mice infected with Δ*sopB* when compared with mice infected with SL1344 (Figure 4B). Moreover, the cytokines production including IFN-γ, CCL2, and CXCL10 were reduced in the cecum of MLKL^−/−^ mice infected with Δ*sopB* when compared with WT mice (Figure 4B). These results indicated that MLKL deletion improved the severity to Δ*sopB* induced colitis. Additionally, we found mucin2 expression in the cecum of MLKL^−/−^ mice infected with Δ*sopB* was increased when compared to WT mice (Figure 4C). Collectively, these data indicated that MLKL mediated necroptosis contributes to the increased severity to colitis in mice infected with Δ*sopB*.

### MLKL Deletion Reduces Bacterial Translocation Following Δ*sopB* Infection

**Figure 5:**
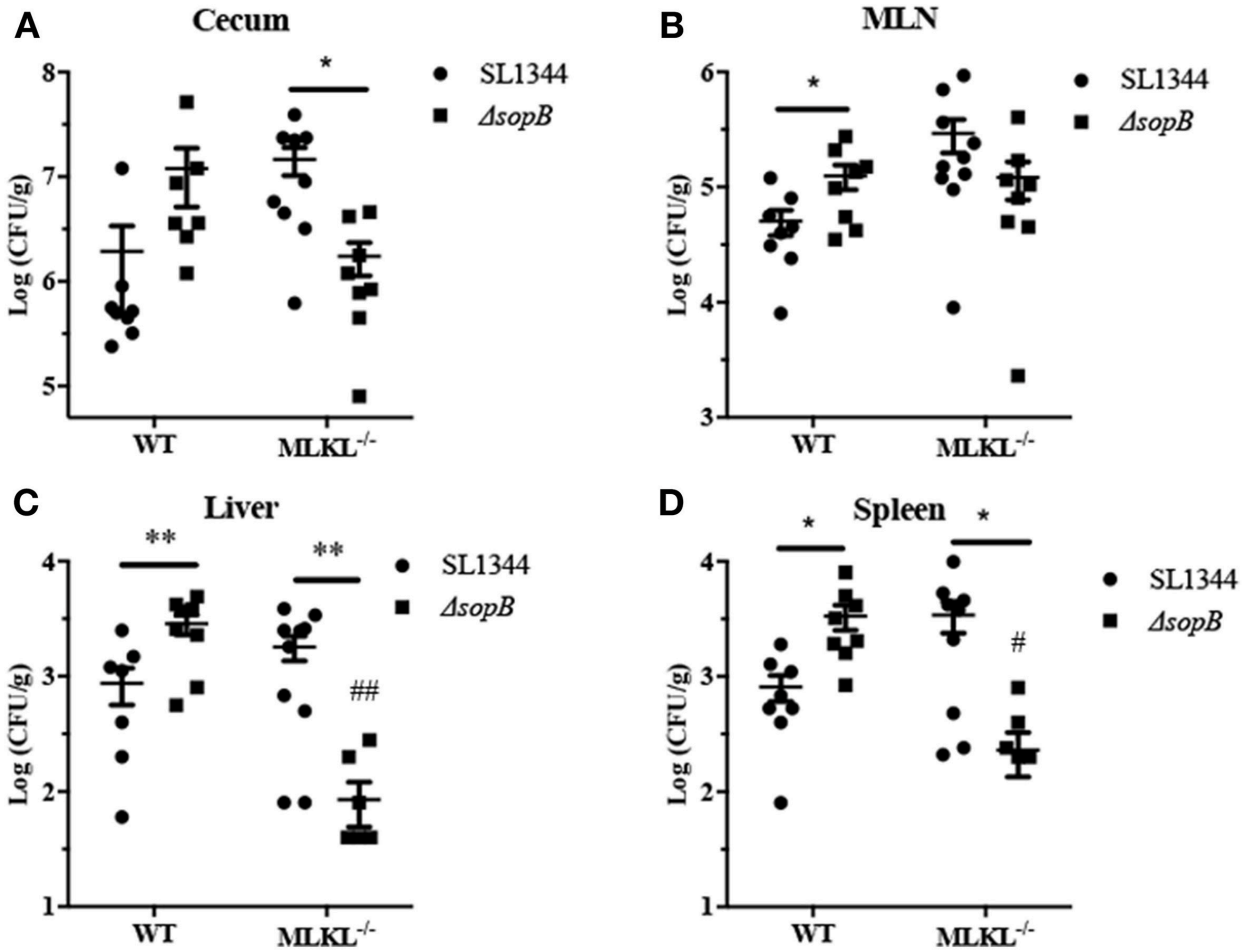
MLKL deletion rescued the increased bacteria burdens in mice infected with Δ*sopB*. WT and MLKL deficient mice were orally administrated with SL1344 or Δ*sopB* for 2 days (*n* = 5), bacterial loads in the cecum **(A)**, MLN **(B)**, liver **(C)**, and spleen **(D)** were determined on day 2 p.i.. *P < 0.05, **P < 0.01, ^#^*P* < 0.05, ^*##*^*P* < 0.01; ^#^stands for comparing with WT mice infected with Δ*sopB*.

*Salmonella* disturbs intestinal barrier and then transmits to systemic sites to establish infection. We subsequently evaluated the effect of Δ*sopB* mediated necroptosis on *Salmonella* translocation. Compared to *Salmonella* infection, Δ*sopB* infected mice had an increased bacterial colonization in cecum (Figure 5A) and significant increased bacterial burdens in mesenteric lymph node (MLN) (Figure 5B), liver (Figure 5C), and spleen (Figure 5D) in WT mice indicating that SopB deletion enhanced *Salmonella* pathogenesis characterized by increased bacterial translocation. However, MLKL deletion abolished the increased bacterial translocation. Following Δ*sopB* infection, MLKL^−/−^ mice had decreased bacterial burdens in liver (Figure 5C) and spleen (Figure 5D) when compared to WT mice. These results indicated that Δ*sopB* infection induced increased necroptosis contributing to the increased bacterial translocation.

### The Level of SopB Expression Is Downregulated During *Salmonella* Infection

**Figure 6:**
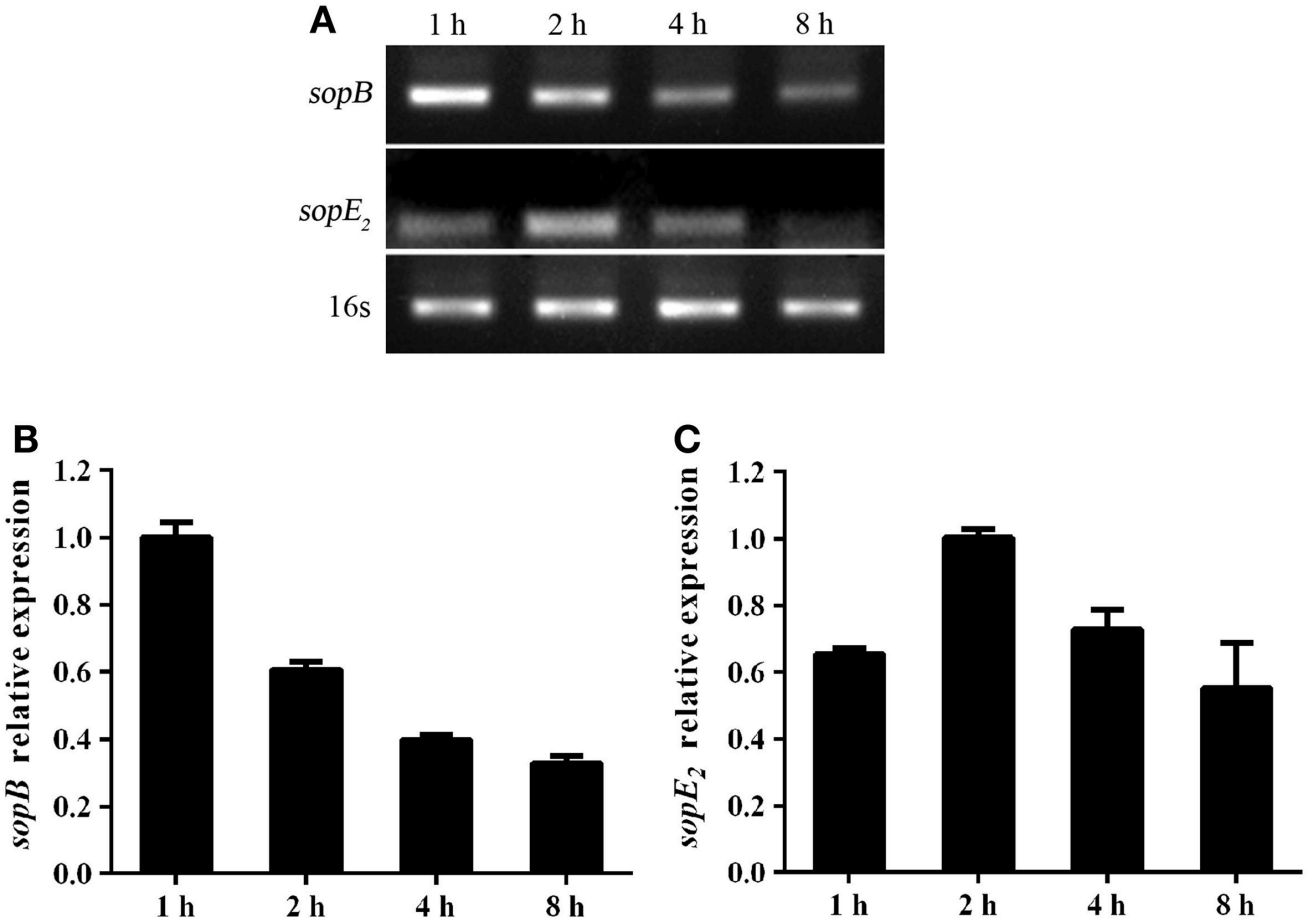
*Salmonella* differently regulated virulence genes expression during infection. Expression of *sopB* **(A,B)** and *sopE*_2_ **(A,C)** mRNA in LS174T cells infected with *Salmonella* for indicated time points were evaluated via RT-PCR.

*Salmonella* is an intracellular bacterial pathogen and has evolved many mechanisms to evade host immune response to facilitate bacteria translocation, such as regulating effector proteins expression. We proved that SopB deletion results in increased cell necroptosis and bacterial translocation. Thus, we hypothesized that *Salmonella* might adoptively downregulate SopB expression to enhance its translocation. Indeed, results showed that the expression level of *sopB* was downregulated in LS174T cells during *Salmonella* infection (Figures 6A,B). The downregulated *sopB* expression would enhance bacteria pathogenesis and increase cell necroptosis which facilitates bacterial translocation. Additionally, we also examined the expression level of SopE_2_ which has a similar function as SopB. In contrast to *sopB*, the expression level of *sopE*_2_ was upregulated in LS174T cells infected with *Salmonella* for 2 h (Figures 6A,C). These results indicated effector proteins were differently regulated during *Salmonella* infection and the downregulated *sopB* expression might represent a strategy used by *Salmonella* to spread to systemic sites and establish infection.

## Discussion

SopB is an important SPI-1 secreted virulence effector and exerts many roles during *Salmonella* infection. However, the mechanisms of SopB in *Salmonella* induced colitis have not been well-studied. To address this, we investigated the severity to colitis following SL1344 or Δ*sopB* infection. Δ*sopB* infection increased goblet cell necroptosis associated with enhanced intestinal inflammation and increased bacterial translocation. The increased goblet cell necroptosis was caused by upregulating MLKL phosphorylation. MLKL deletion rescued the intestinal inflammation and bacterial loads in mice infected with Δ*sopB*. Interestingly, SopB expression was downregulated during *Salmonella* infection. The down regulated SopB would increase cell necroptosis similar to SopB deletion which enable bacteria escape from the epithelial cell to infect neighbor cells and consequently translocated to systemic sites. Collectively, this study indicated a functional role for SopB in preventing cell necroptosis and the downregulation of SopB expression represents a mechanism used by *Salmonella* to manipulate onset of epithelial cell death to establish infection.

*Salmonella* infection induced cell death is considered to be a major course of pathogenesis of gut inflammation and is tightly controlled. Many effectors translocated into epithelial cells involve in modulating cell death. It has been suggested that the effector protein SopB protects cell from apoptosis and pyroptosis (Knodler et al., 2005; Hu et al., 2017). In our study, SopB was additionally shown to prevent goblet cell necroptosis. We found Δ*sopB* infection increased goblet cell necroptosis in cecum via upregulating MLKL phosphorylation. Consistently, Δ*sopB* infection increased LS174T cell death, whereas NSA treatment rescued the increased LS174T cell death induced by Δ*sopB*. These results indicated that SopB play a role in preventing cell necroptosis. However, NSA treatment did not absolutely abolish the increased cell death mediated by Δ*sopB*. This might cause by that SopB can regulate cell death in a manner different from necroptosis. Consistent with a recent study that Δ*sopB* infection induced increased numbers of caspase-3 and caspase-8 positive cells, while a similar phenotype was not observed after Δ*sopBE*_2_ infection indicated that SopB counteracts the proapoptotic effect mediated by SopE_2_ in an undefined mechanism (Zhang et al., 2018). Intriguingly, we found the expression of SopE_2_ in LS174T cell were increased after Δ*sopB* infection for 2 h which might promote cell apoptosis and contribute to the increased numbers of caspase3 and caspase-8 positive cells induced by Δ*sopB* infection.

Necroptosis is generally considered to be pro-inflammatory and has a role in pathogenesis of bacterial infection (Kitur et al., 2015; Pasparakis and Vandenabeele, 2015; Parker and Prince, 2016). The hall mark of necroptosis is that MLKL forms pores or actives ion channels in the membrane of cells resulting in membrane rupture and the leakage of intracellular contents such as inflammatory mediators (Jorgensen et al., 2017). Due to overt inflammatory response caused by the leakage of immunostimulatory contents, necroptosis is considered to be an inflammatory death (Newton and Manning, 2016). Inflammation is required for the control of bacterial infection. However, the overt and/or prolonged inflammation is one of the reasons for local tissue pathogenesis contributing to the excessive bacterial translocation and the development of pathogenesis of inflammatory disease. Additionally, epithelial cell necroptosis facilitates bacteria escape host cell and translocate to systemic sites. Numerous studies have demonstrated that necroptosis has a crucial role in enhancing infection (Robinson et al., 2012; Kitur et al., 2015). Studies have shown that *Salmonella* exploits type I IFN signaling to induce macrophage necroptosis (Robinson et al., 2012; Hu and Zhao, 2013). *Ifnar*^−/−^ mice challenged with *Salmonella* showed an improved survival as well as lower bacterial burdens in liver and spleen. Inhibition of necroptosis induced by *Salmonella* infection reduced inflammation and improve bacterial clearance (Robinson et al., 2012). Consistently, in present study we found Δ*sopB* infection induced an increased level of MLKL phosphorylation in cells of cecum associated with increased inflammation and bacterial translocation. Deletion the final executor of necroptosis MLKL abolished the increased bacterial translocation and severity to inflammation following Δ*sopB* infection. All these results indicated necroptosis represents a pro-inflammatory response. The increased epithelial cell necroptosis mediated by Δ*sopB* infection is a mechanism used by *Salmonella* to breakdown epithelial barrier and spread to systemic sites. However, we did not consider the potential effect of MLKL deficiency on intestinal microbiota composition, which may affect intestinal disease symptoms. Recently, a newly published study found MLKL deficiency has a little effect on intestinal microbiota composition which did not affect severity to DSS induced colitis (Zhang et al., 2019). Whether the altered intestinal microbiota contributes to *Salmonella* induced disease need to be further investigated.

To establish an infection, *Salmonella* first enters into epithelial cell and replicates within *Salmonella*-containing vacuole, and then escapes from epithelial cell in the help of effectors (Fabrega and Vila, 2013). Once the epithelial barrier is disrupted, *Salmonella* is internalized by macrophage and subsequently transmits to systemic sites. All these phases are connected with cell death. In the initial stage of infection, cell death must be prevented which provides a favorite environment for *Salmonella* replication. During the last phage of infection, cell death is required to promote *Salmonella* escape epithelial cells. To realize this role switching, effector proteins are temporally regulated. In present study, we found SopB expression was downregulated. The downregulated SopB would induce cell necroptosis which potentially switches the role of SopB from epithelial cell invasion to escape host cells. Therefore, the temporally regulated SopB secretion represents a virulence strategy used by *Salmonella* to establish infection. Epithelial cells have important roles in protecting enteric pathogens infection (Broz et al., 2012). SopB cooperates with other effectors to mediate bacterial invasion and cell death. Though SopB is required for invasion, deletion of SopB fail to alter the ability of invasion and has no pathogenesis defect. Additionally, we found SopB not only mediated the upregulation of MLKL phosphorylation in epithelial cell, but also in immune cells. However, we focus the function of SopB in goblet cells due to the mainly difference in pathogenesis as well as the crucial role of goblet cell in defending against enteric pathogens infection.

In addition to the role in delaying apoptosis and pyroptosis, this study indicated a different role for SopB in modulating cell necroptosis. We found SopB deletion upregulated MLKL phosphorylation which increased cell necroptosis and consequently increased severity to colitis and promotes bacterial translocation. This study further identified a different biological mechanism that the downregulated SopB expression increased host cell necroptosis represents a devised strategy used by *Salmonella* to establish infection.

## Author Contributions

G-QH and Y-JY performed most of the experiments, collected and analyzed the results, and wrote the manuscript. WC conceived and designed the study. X-XQ, SQ, and S-XY performed the animal study. JZ performed the IHC experiments. C-TD performed the H&E study. All authors have read the manuscript and approved the final manuscript.

### Conflict of Interest Statement

The authors declare that the research was conducted in the absence of any commercial or financial relationships that could be construed as a potential conflict of interest.

## References

[B1] Abrahams G. L. Hensel M. (2006). Manipulating cellular transport and immune responses: dynamic interactions between intracellular *Salmonella enterica* and its host cells. Cell. Microbiol. 8, 728–737. 10.1111/j.1462-5822.2006.00706.x 16611223

[B2] Barthel M. Hapfelmeier S. Quintanilla-Martinez L. Kremer M. Rohde M. Hogardt M.. (2003). Pretreatment of mice with streptomycin provides a *Salmonella enterica* serovar Typhimurium colitis model that allows analysis of both pathogen and host. Infect Immun 71, 2839–2858. 10.1128/IAI.71.5.2839-2858.2003 12704158 PMC153285

[B3] Bergstrom K. S. Kissoon-Singh V. Gibson D. L. Ma C. Montero M. Sham H. P.. (2010). Muc2 protects against lethal infectious colitis by disassociating pathogenic and commensal bacteria from the colonic mucosa. PLoS Pathog. 6:e1000902. 10.1371/journal.ppat.1000902 20485566 PMC2869315

[B4] Bertelsen L. S. Paesold G. Marcus S. L. Finlay B. B. Eckmann L. Barrett K. E. (2004). Modulation of chloride secretory responses and barrier function of intestinal epithelial cells by the Salmonella effector protein SigD. Am. J. Physiol. Cell. Physiol. 287, C939–948. 10.1152/ajpcell.00413.2003 15175224

[B5] Birchenough G. M. Nystrom E. E. Johansson M. E. Hansson G. C. (2016). A sentinel goblet cell guards the colonic crypt by triggering Nlrp6-dependent Muc2 secretion. Science 352, 1535–1542. 10.1126/science.aaf7419 27339979 PMC5148821

[B6] Broz P. Newton K. Lamkanfi M. Mariathasan S. Dixit V. M. Monack D. M. (2010). Redundant roles for inflammasome receptors NLRP3 and NLRC4 in host defense against Salmonella. J. Exp. Med. 207, 1745–1755. 10.1084/jem.20100257 20603313 PMC2916133

[B7] Broz P. Ohlson M. B. Monack D. M. (2012). Innate immune response to *Salmonella typhimurium*, a model enteric pathogen. Gut Microbes 3, 62–70. 10.4161/gmic.19141 22198618 PMC3370950

[B8] Cooper K. G. Winfree S. Malik-Kale P. Jolly C. Ireland R. Knodler L. A.. (2011). Activation of Akt by the bacterial inositol phosphatase, SopB, is wortmannin insensitive. PLoS ONE 6:e22260. 10.1371/journal.pone.0022260 21779406 PMC3136525

[B9] Fabrega A. Vila J. (2013). *Salmonella enterica* serovar Typhimurium skills to succeed in the host: virulence and regulation. Clin. Microbiol. Rev. 26, 308–341. 10.1128/CMR.00066-12 23554419 PMC3623383

[B10] Galan J. E. (2001). Salmonella interactions with host cells: type III secretion at work. Annu. Rev. Cell Dev. Biol. 17, 53–86. 10.1146/annurev.cellbio.17.1.53 11687484

[B11] Giacomodonato M. N. Sarnacki S. H. Llana M. N. Cerquetti M. C. (2011). SopB effector protein of *Salmonella typhimurium* is translocated in mesenteric lymph nodes during murine salmonellosis. FEMS Microbiol. Lett. 317, 100–106. 10.1111/j.1574-6968.2011.02217.x 21241360

[B12] Hefele M. Stolzer I. Ruder B. He G. W. Mahapatro M. Wirtz S.. (2018). Intestinal epithelial Caspase-8 signaling is essential to prevent necroptosis during *Salmonella typhimurium* induced enteritis. Mucosal Immunol. 11, 1191–1202. 10.1038/s41385-018-0011-x 29520026

[B13] Hensel M. (2004). Evolution of pathogenicity islands of *Salmonella enterica*. Int. J. Med. Microbiol. 294, 95–102. 10.1016/j.ijmm.2004.06.025 15493819

[B14] Hu G. Q. Song P. X. Chen W. Qi S. Yu S. X. Du C. T.. (2017). Cirtical role for Salmonella effector SopB in regulating inflammasome activation. Mol. Immunol. 90, 280–286. 10.1016/j.molimm.2017.07.011 28846926

[B15] Hu Z. Q. Zhao W. H. (2013). Type 1 interferon-associated necroptosis: a novel mechanism for *Salmonella enterica* Typhimurium to induce macrophage death. Cell. Mol. Immunol. 10, 10–12. 10.1038/cmi.2012.54 23147719 PMC4003180

[B16] Jorgensen I. Rayamajhi M. Miao E. A. (2017). Programmed cell death as a defence against infection. Nat. Rev. Immunol. 17, 151–164. 10.1038/nri.2016.147 28138137 PMC5328506

[B17] Kirk M. D. Pires S. M. Black R. E. Caipo M. Crump J. A. Devleesschauwer B. (2015). World Health Organization estimates of the global and regional disease burden of 22 foodborne bacterial, protozoal, and viral diseases, 2010: a data synthesis. PLoS Med. 12:e1001921 10.1371/journal.pmed.1001921 26633831 PMC4668831

[B18] Kitur K. Parker D. Nieto P. Ahn D. S. Cohen T. S. Chung S.. (2015). Toxin-induced necroptosis is a major mechanism of Staphylococcus aureus lung damage. PLoS Pathog. 11:e1004820. 10.1371/journal.ppat.1004820 25880560 PMC4399879

[B19] Knodler L. A. Finlay B. B. Steele-Mortimer O. (2005). The Salmonella effector protein SopB protects epithelial cells from apoptosis by sustained activation of Akt. J. Biol. Chem. 280, 9058–9064. 10.1074/jbc.M412588200 15642738

[B20] Kum W. W. Lo B. C. Yu H. B. Finlay B. B. (2011). Protective role of Akt2 in *Salmonella enterica* serovar typhimurium-induced gastroenterocolitis. Infect. Immun. 79, 2554–2566. 10.1128/IAI.01235-10 21555401 PMC3191998

[B21] McGhie E. J. Brawn L. C. Hume P. J. Humphreys D. Koronakis V. (2009). Salmonella takes control: effector-driven manipulation of the host. Curr. Opin. Microbiol. 12, 117–124. 10.1016/j.mib.2008.12.001 19157959 PMC2647982

[B22] Newton K. Manning G. (2016). Necroptosis and inflammation. Annu. Rev. Biochem. 85, 743–763. 10.1146/annurev-biochem-060815-014830 26865533

[B23] Parker D. Prince A. (2016). Immunoregulatory effects of necroptosis in bacterial infections. Cytokine 88, 274–275. 10.1016/j.cyto.2016.09.024 27710879 PMC5067227

[B24] Pasparakis M. Vandenabeele P. (2015). Necroptosis and its role in inflammation. Nature 517, 311–320. 10.1038/nature14191 25592536

[B25] Pelaseyed T. Bergstrom J. H. Gustafsson J. K. Ermund A. Birchenough G. M. Schutte A.. (2014). The mucus and mucins of the goblet cells and enterocytes provide the first defense line of the gastrointestinal tract and interact with the immune system. Immunol. Rev. 260, 8–20. 10.1111/imr.12182 24942678 PMC4281373

[B26] Piscatelli H. L. Li M. Zhou D. (2016). Dual 4- and 5-phosphatase activities regulate SopB-dependent phosphoinositide dynamics to promote bacterial entry. Cell. Microbiol. 18, 705–719. 10.1111/cmi.12542 26537021

[B27] Raymond B. Young J. C. Pallett M. Endres R. G. Clements A. Frankel G. (2013). Subversion of trafficking, apoptosis, and innate immunity by type III secretion system effectors. Trends Microbiol. 21, 430–441. 10.1016/j.tim.2013.06.008 23870533

[B28] Robinson N. McComb S. Mulligan R. Dudani R. Krishnan L. Sad S. (2012). Type I interferon induces necroptosis in macrophages during infection with *Salmonella enterica* serovar Typhimurium. Nat. Immunol. 13, 954–962. 10.1038/ni.2397 22922364 PMC4005791

[B29] Ruan H. Zhang Z. Tian L. Wang S. Hu S. Qiao J. J. (2016). The Salmonella effector SopB prevents ROS-induced apoptosis of epithelial cells by retarding TRAF6 recruitment to mitochondria. Biochem. Biophys. Res. Commun. 478, 618–623. 10.1016/j.bbrc.2016.07.116 27473656

[B30] Schmidt H. Hensel M. (2004). Pathogenicity islands in bacterial pathogenesis. Clin. Microbiol. Rev. 17, 14–56. 10.1128/CMR.17.1.14-56.2004 14726454 PMC321463

[B31] Stecher B. Macpherson A. J. Hapfelmeier S. Kremer M. Stallmach T. Hardt W. D. (2005). Comparison of *Salmonella enterica* serovar Typhimurium colitis in germfree mice and mice pretreated with streptomycin. Infect. Immun. 73, 3228–3241. 10.1128/IAI.73.6.3228-3241.2005 15908347 PMC1111827

[B32] Tobar J. A. Carreno L. J. Bueno S. M. Gonzalez P. A. Mora J. E. Quezada S. A.. (2006). Virulent *Salmonella enterica* serovar typhimurium evades adaptive immunity by preventing dendritic cells from activating T cells. Infect. Immun. 74, 6438–6448. 10.1128/IAI.00063-06 17057096 PMC1695529

[B33] Zhang J. Qin D. Yang Y. J. Hu G. Q. Qin X. X. Du C. T.. (2019). MLKL deficiency inhibits DSS-induced colitis independent of intestinal microbiota. Mol. Immunol. 107, 132–141. 10.1016/j.molimm.2019.01.018 30738250

[B34] Zhang K. Riba A. Nietschke M. Torow N. Repnik U. Putz A.. (2018). Minimal SPI1-T3SS effector requirement for Salmonella enterocyte invasion and intracellular proliferation *in vivo*. PLoS Pathog. 14:e1006925. 10.1371/journal.ppat.1006925 29522566 PMC5862521

